# Associations of dietary habits and meal regularity with gastrointestinal symptoms, depression, and quality of life in patients with inflammatory bowel disease: a cross-sectional study

**DOI:** 10.1186/s12876-026-04999-9

**Published:** 2026-06-17

**Authors:** Elif Nur Özen, Ali İbrahim Hatemi

**Affiliations:** 1 Institute of Graduate Studies, Department of Internal Medicine, Nutrition (Internal Diseases), Istanbul University-Cerrahpaşa, Istanbul, Turkey; 2 Faculty of Health Sciences, Department of Nutrition and Dietetics, Istinye University, Istanbul, Turkey; 3 Cerrahpaşa Medical Faculty, Department of Internal Medicine, Division of Gastroenterology, Istanbul University-Cerrahpaşa, Istanbul, Turkey

**Keywords:** Crohn’s Disease, Diet, Inflammatory Bowel Disease, Nutrition, Ulcerative Colitis

## Abstract

**Background:**

Inflammatory Bowel Disease (IBD) is a chronic and recurrent gastrointestinal disorder that adversely affects patients’ quality of life. The influence of daily dietary behaviors and meal regularity on gastrointestinal symptoms and quality of life remains under-investigated. The study aimed to evaluate dietary intake, dietary habits, and meal patterns of the IBD patients and based on this data, to determine their gastrointestinal symptoms, quality of life, and depression status.

**Methods:**

This cross-sectional study was conducted at Istanbul University Cerrahpaşa Medical Faculty Hospital and included adults aged 18–75 years with a confirmed diagnosis of IBD attending routine outpatient follow-up visits. Data were collected using a sociodemographic and clinical data form, a 24-Hour Dietary Recall, IBD Quality of Life Questionnaire (IBD-QoL), Beck Depression Inventory II (BDI-II), Gastrointestinal Symptom Rating Scale (GSRS).

**Results:**

A total of 50 patients with IBD were included (30 Crohn’s disease and 20 Ulcerative colitis). The mean age was 39.5 ± 13.49 years and the mean BMI was 24.82 ± 5.39 kg/m². Most patients (82%) reported consuming foods that worsened their symptoms, primarily legumes (21%), milk (14.3%), and raw vegetables (10.9%). Conversely, 50% identified foods that alleviated symptoms, mainly yogurt (18.2%) and cooked vegetables (18.2%), cooked meat (7.6%). Dietary analysis revealed intake below recommended levels for fiber, vitamins B1, B2, B6, C, folate, potassium, calcium, magnesium, and iron. Meal skipping was reported by 20% of patients with Ulcerative colitis and 40% with Crohn’s disease. Patients who skipped meals had higher gastrointestinal symptom scores than those with regular meal patterns. Additionally, higher depression scores were associated with lower quality of life and more severe gastrointestinal symptoms.

**Conclusion:**

The study found that dietary intake and meal patterns in individuals with IBD are associated with their quality of life and gastrointestinal symptoms. A balanced and regular diet may be associated with reduced gastrointestinal symptoms and improved quality of life in IBD patients.

**Supplementary Information:**

The online version contains supplementary material available at 10.1186/s12876-026-04999-9.

## Introduction

 Inflammatory Bowel Disease (IBD) is a chronic inflammatory condition characterized by abdominal pain, chronic diarrhea and extraintestinal manifestations which can be divided into two major phenotypes: Crohn’s Disease (CD) and Ulcerative Colitis (UC) [1, 2]. Although its etiology and pathogenesis are not fully understood, IBD is considered multifactorial, arising from a complex interplay between genetic susceptibility, environmental exposures, and dysregulated immune responses [3, 4]. Dysregulation of the innate and adaptive immune systems and impairment of intestinal epithelial barrier integrity through tight junction disruption have been implicated in the initiation and exacerbation of IBD. In addition, dietary factors and various environmental exposures –including medications, infections, pollution, psychosocial stressors, smoking, and alcohol consumption– may further modulate immune responses and influence disease progression [5, 6].

UC and CD differ markedly in terms of disease location, depth of inflammation, and pattern of disease extension. UC is a chronic inflammatory condition that begins in the rectum, progresses proximally in a continuous manner, and primarily involves the mucosal layer, whereas CD is characterized by transmural inflammation and most commonly affects the terminal ileum and colon; however, it may involve any part of the gastrointestinal tract from the mouth to the anus [7–9]. Symptoms of both conditions can range from mild to severe and may even be life-threatening [8]. Modifiable lifestyle factors, particularly diet, can alter the composition and function of gut microbiota, thereby regulating the mucosal immune system and influencing disease development [10–12].

Nutrition plays a crucial role in maintaining the homeostasis of the intestinal microenvironment by affecting microbial composition and function, the intestinal barrier, host immunity, and gut physiology [13–15]. The intestinal barrier, composed of tightly connected cells and immune system cells forming a mucosal layer, serves as the first line of defense against bacteria, pathogens, and dietary antigens. Dietary factors can disrupt this barrier, leading to increased intestinal permeability and subsequent inflammatory changes [16, 17].

Globalization, industrialization, urbanization, and Westernized dietary patterns, along with lifestyle changes, have contributed to the rising incidence and prevalence of IBD, particularly in developing countries [7, 14, 18, 19].

**Fig. 1:**
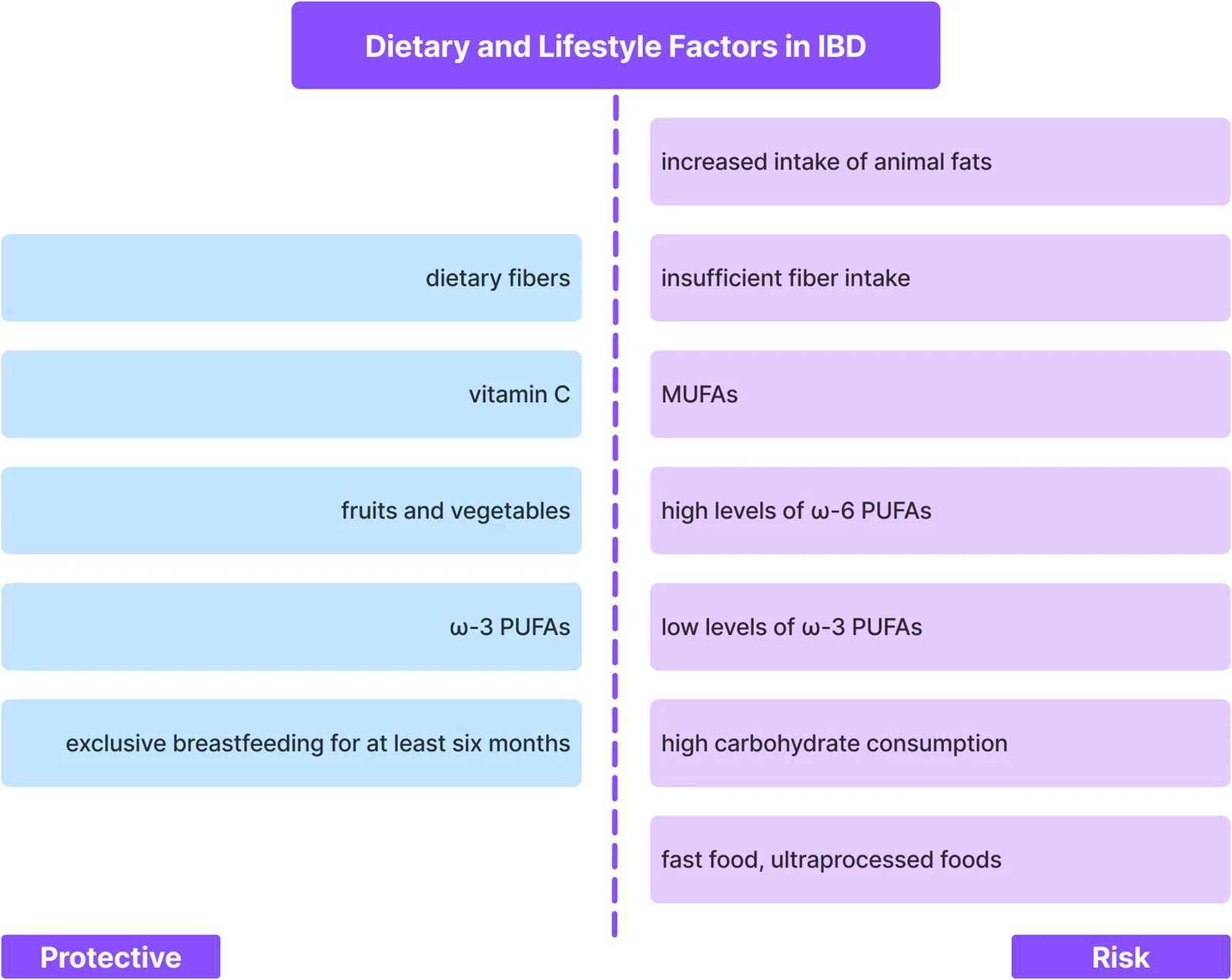
Dietary and Lifestyle Factors in IBD

As shown in Fig. 1, increased intake of animal fats, monounsaturated fatty acids (MUFAs), high levels of ω-6 polyunsaturated fatty acids (PUFAs), low levels of ω-3 PUFAs, fast food, high carbohydrate consumption, and insufficient fiber intake are considered risk factors for IBD. Conversely, dietary fibers, vitamin C, fruits and vegetables, ω-3 PUFAs, and exclusive breastfeeding for at least six months serve as protective factors against IBD [9, 14, 20, 21]. A dietary plan characterized by high meat and fat content but low fiber content can lead to thinning of the mucosal layer, excessive bacterial proliferation, and increased intestinal permeability [16, 22].

The ideal goal in the treatment of IBD is to achieve deep remission, defined as a combination of symptomatic remission and endoscopic improvement [23, 24]. While there is no specific diet tailored to the disease, a common recommendation is to avoid processed foods, products rich in additives, and those high in saturated and trans fatty acids [25, 26]. Identifying dietary patterns that influence disease activity can be effectively achieved through detailed patient interviews, as individuals are often better able to recognize foods that exacerbate rather than alleviate their symptoms [27]. Screening for macro- and micronutrient deficiencies is essential, particularly in patients who are malnourished or at risk of malnutrition [7]. Foods that are avoided should be evaluated, healthy eating should be encouraged, long-term restrictions should be avoided, dietary variety should be promoted, a refeeding phase following exclusion diets should be planned, and supplements should be provided to prevent nutrient deficiencies. Multidisciplinary collaboration is crucial in disease management [27].

Given the clinical importance of nutrition in IBD management [13, 14, 16], the present study evaluated dietary intake, dietary habits, and meal patterns in patients with IBD and examined their associations with gastrointestinal symptoms, quality of life, and depression.

## Methods

### Study design and aim

This cross-sectional study enrolled patients with IBD to comprehensively assess their dietary intake, dietary habits, and meal patterns, while exploring associations with gastrointestinal symptoms, quality of life, and depression levels. Given the cross-sectional design, all findings are associative and do not imply causality.

### Patient population

**Fig. 2:**
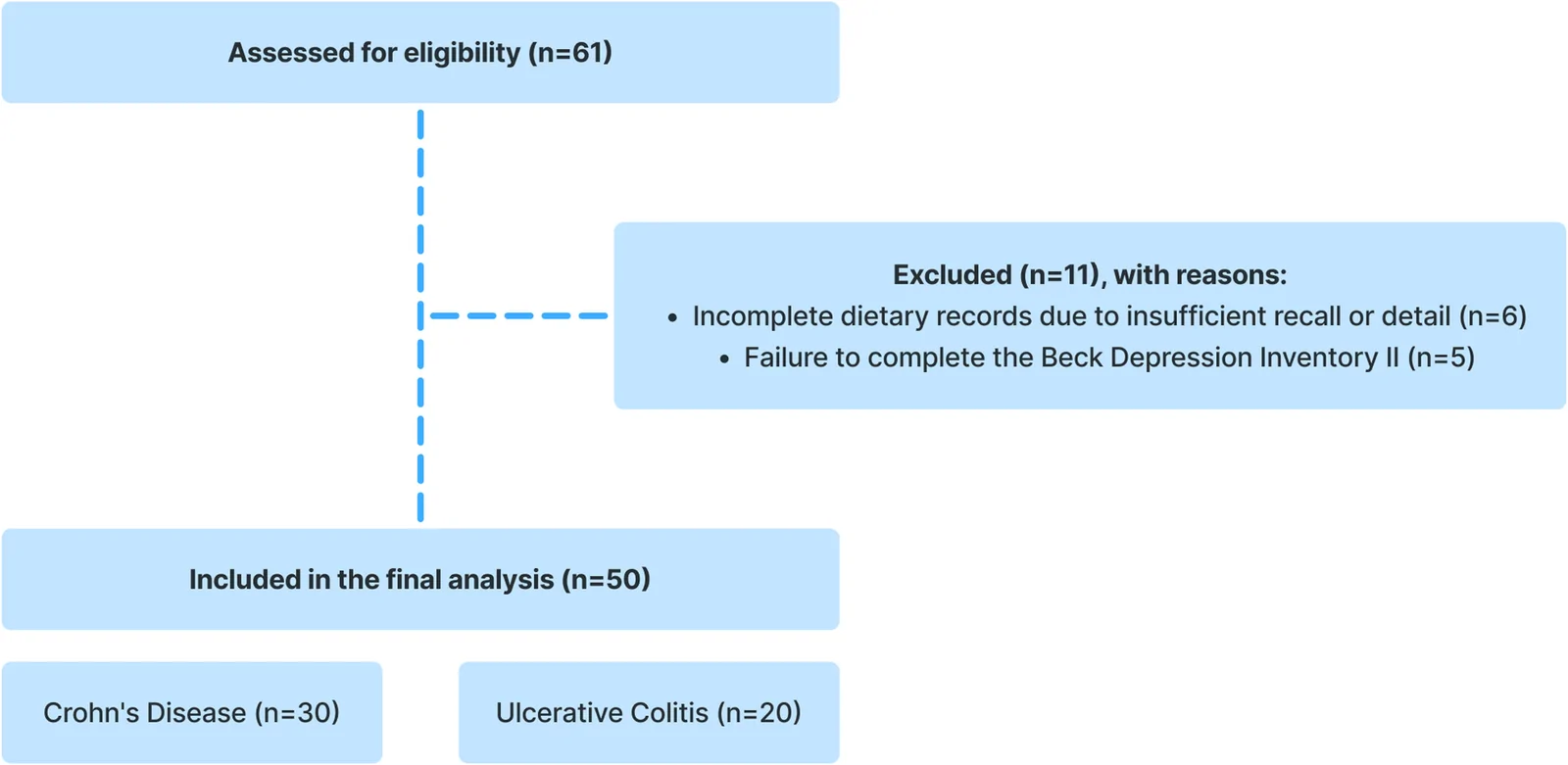
Flow Diagram

The recruitment process and the distribution of participants are detailed in the participant flow diagram (Fig. 2). Initially, 61 patients were assessed for eligibility; however, 11 patients were excluded due to incomplete dietary records resulting from insufficient recall or lack of detail (*n* = 6), as well as failure to complete the Beck Depression Inventory II (*n* = 5; due to participant reluctance to provide psychosocial data), resulting in a final study population of 50 individuals. This final sample size was justified by a power analysis conducted with a two-tailed alpha level of 0.05 and a statistical power of 90% (β = 0.10). Based on detecting a moderate effect size (*r* = 0.40) for the association between dietary variables and gastrointestinal symptom scores, the minimum required sample size was determined to be 43 participants; thus, the inclusion of 50 individuals ensured the study was sufficiently powered while accounting for potential dropouts or incomplete data.

Participants were adults aged 18–75 years with a previously established diagnosis of IBD, confirmed through standard clinical, endoscopic, and histopathological criteria, who attended routine outpatient follow-up visits at the Istanbul University Cerrahpaşa Medical Faculty Gastroenterology outpatient clinic between August and September 2023. To specifically examine the influence of patient-reported dietary behaviors and meal regularity –aspects often overshadowed by clinical parameters in IBD management– the study primarily focused on dietary behaviors and meal patterns. Consequently, participants were recruited regardless of disease activity status, as no formal disease activity assessment was performed. Therefore, adjustment for disease severity in statistical analyses was not possible. Similarly, data regarding specific medication use (e.g., biologics, steroids, or immunomodulators) were not systematically recorded; these omissions are addressed in the limitations section. Inclusion criteria required the ability to communicate effectively and make independent dietary choices. Individuals with psychiatric disorders, or those who were pregnant or breastfeeding, were excluded.

The study was approved by the Ethics Committee of Non-Invasive Clinical Trials at Istanbul University Cerrahpaşa (decision number 754308, dated August 8, 2023). All participants were provided with comprehensive information about the purpose and duration of the study, and those who agreed to participate signed an informed consent form. The research was conducted in accordance with the Declaration of Helsinki.

### Data collection tools

A structured sociodemographic and clinical data form was administered to voluntary participants to collect demographic information and assess dietary habits, including age, gender, education level, duration of diagnosis, number of daily meals, and frequency of meal skipping. The anthropometric measurements section included two self-reported items on height and body weight; Body Mass Index (BMI) was calculated by the researchers based on these measurements. A 24-Hour Dietary Recall was administered as a face-to-face interview to determine the foods consumed and daily energy and nutrient intakes; portion sizes were estimated using standard household measures. Dietary data were analyzed using the BeBiS dietary analysis software (Nutrition Information Systems, Turkey) [28] and compared against reference values recommended by the European Food Safety Authority (EFSA) [29]. Dietary intake of each nutrient was compared against the EFSA Dietary Reference Values for the respective age and gender groups. Nutrients falling below the recommended intake levels were identified and reported. Foods perceived to influence gastrointestinal symptoms were assessed using two structured interview questions regarding foods that alleviate or exacerbate symptoms. Responses were self-reported and categorized into food groups for analysis; this was not based on a validated questionnaire. The Inflammatory Bowel Disease Quality of Life Questionnaire (IBD-QoL), comprising 36 items, was utilized to assess quality of life, evaluating systemic symptoms, bowel symptoms, functional impairment, social impairment, and emotional function [30, 31]. The Beck Depression Inventory II (BDI-II), a 21-item questionnaire measuring characteristic attitudes and symptoms of depression, was used to assess depression status [32, 33]. To measure gastrointestinal symptoms, the Gastrointestinal Symptom Rating Scale (GSRS) consisting of 15 items across five subcategories (abdominal pain syndrome, reflux syndrome, diarrhea syndrome, indigestion syndrome, and constipation syndrome), was used [34, 35].

### Statistical analysis

All statistical analyses were performed using IBM SPSS version 25.0 (SPSS Inc., Chicago, Illinois, USA). Continuous variables are presented as median (min-max) and mean ± standard deviation (SD) in the tables. Categorical variables are presented as frequency (n) and percentage (%). When examining the continuous data of the study for normality assumptions, it was determined that they did not demonstrate a normal distribution, as indicated by Shapiro-Wilk values (*p* < 0.05) and the values obtained from dividing Skewness and Kurtosis by their standard errors, which fell outside the ± 1.96 threshold range [36, 37]. The Spearman correlation test was utilized to determine the relationship between continuous variables. For two-group variables, the Mann-Whitney U test was applied, while the Kruskal-Wallis H test was used for variables with three or more groups. In cases where a significant difference was found between groups, the Games-Howell post-hoc test was preferred to determine which groups differed. A p-value of < 0.05 was considered statistically significant. Due to the absence of disease activity measures and the limited sample size, multivariate regression analyses were not performed.

## Results

A total of 50 patients diagnosed with IBD were included in the study (30 CD, 20 UC). Among them, 42.0% (*n* = 21) were female and 58.0% (*n* = 29) were male. The mean age was 39.5 ± 13.49 years, and the mean BMI was 24.82 ± 5.39 kg/m² (Table 1). About 44.0% (*n* = 22) of patients had a chronic disease other than IBD. A total of 20.0% (*n* = 10) had received nutrition education, 4.0% (*n* = 2) were on a specific diet, and 50.0% (*n* = 25) reported using vitamin and mineral supplements, with Vitamin D (28.6%, *n* = 18), iron (12.7%, *n* = 8), and Vitamin B12 (7.9%, *n* = 5) being the most common (Table 2).

**Table 1:** Descriptive statistics of patients’ age and anthropometric measurements

Variables	*n* or Median (Min-Max)	% or Mean ± SD
Age (years)	39,5 (19,0–71,0)	39,5 ± 13,49
Heights (m)	1,70 (1,55 − 1,90)	1,69 ± 0,08
Body Weight (kg)	70,0 (41,0-108,0)	70,94 ± 14,76
BMI (kg / m^2^)	24,24 (16,02–42,19)	24,82 ± 5,39
Underweight (< 18,5 kg / m^2^)	5	10,0
Normal weight (18,5–24,9 kg / m^2^)	23	46,0
Overweight (25,0–29,9 kg / m^2^)	13	26,0
Obese (≥ 30 kg / m^2^)	9	18,0

The minimum age of the participants was 19 years, the maximum age was 71 years, with a median age of 39.5 years. The average age was also 39.5 years (SD = 13.49). The average height of the participants was 1.69 m (min = 1.55, max = 1.90, median = 1.70), the average body weight was 70.94 kg (min = 41.0, max = 108.0, median = 70.0), and the average BMI was 24.82 (min = 16.02, max = 42.19, median = 24.24). It was determined that 10.0% (*n* = 5) of the participants were underweight, 46.0% (*n* = 23) were normal weight, 26.0% (*n* = 13) were overweight, and 18.0% (*n* = 9) were obese (Table 2)

n= Number, %= Percentage, Min= Minimum, Max= Maximum, SD = Standard Deviation

**Table 2:** Descriptive Characteristics of IBD Patients

Variables	IBD Diagnosis		
	Crohn’s Disease (*n* = 30)	Ulcerative Colitis (*n* = 20)	*p*
Gender			
Female	10 (33,3)	11 (55)	0.128^a^
Male	20 (66,7)	9 (45)	
Type of Chronic Disease*			
None	15 (42,9)	13 (56,5)	**-**
Cardiovascular diseases	1 (2,9)	1 (4,3)	
Diabetes	-	1 (4,3)	
Hypertension	3 (8,6)	-	
Cancer	1 (2,9)	-	
Endocrine diseases	1 (2,9)	2 (8,7)	
Musculoskeletal problems	5 (14,3)	1 (4,3)	
Respiratory system diseases	2 (5,7)	1 (4,3)	
Gastrointestinal system diseases	3 (8,6)	4 (17,4)	
Other	4 (11,5)	-	
IBD Diagnosis Duration			0.280^b^
Less than 1 year	0 (0)	2 (10)	
1–3 years	6 (20)	2 (10)	
3–5 years	9 (30)	6 (30)	
5–10 years	7 (23,3)	7 (35)	
More than 10 years	8 (26,7)	3 (15)	
Has the patient received nutrition education?			
Yes	7 (23,3)	3 (15)	0.470^b^
No	23 (76,7)	17 (85)	
Where did the patient receive nutrition education?			
No	23 (76,7)	17 (85)	0.388^b^
Dietitian	5 (16,7)	1 (5)	
Physician	2 (6,7)	1 (5)	
Media	0 (0)	1 (5)	
Is the patient following a specific diet?			
Yes	2 (6,7)	0 (0)	0.239^b^
No	28 (93,3)	20 (100)	
If following a diet, what type?			
None	28 (93,3)	20 (100)	0.499^b^
Protein-based	1 (3,3)	0 (0)	
Gluten-free	1 (3,3)	0 (0)	
Is the patient using any vitamins or minerals?			
Yes	14 (46,7)	11 (55)	0.564^b^
No	16 (53,3)	9 (45)	
If using vitamins or minerals, which ones? *			
No	18 (47,4)	7 (28,0)	-
Vitamin D	10 (26,39	8 (32,0)	
Vitamin B12	2 (5,3)	3 (12,0)	
Vitamin C	1 (2,6)	-	
Folic acid	1 (2,6)	-	
Iron	3 (7,9)	5 (20,0)	
Magnesium	-	1 (4,0)	
Calcium	2 (5,3)	-	
Multivitamin	1 (2,6)	1 (4,0)	
Beck Depression Inventory II Scoring			
Minimal	22 (73,3)	17 (85)	0.442^b^
Mild	6 (20)	1 (5)	
Moderate	2 (6,7)	2 (10)	

Among the 50 IBD patients, including 20 UC and 30 with CD, 42.0% (*n* = 21) were female, and 58.0% (*n* = 29) were male. It was found that 44.0% (*n* = 22) of the participants had a chronic illness other than IBD. The majority of the participants, 30.0% (*n* = 15), had a diagnosis duration of 3–5 years. Additionally, 20.0% (*n* = 10) of the participants received nutrition education, 12.0% (*n* = 6) received education from a dietitian, 4.0% (*n* = 2) were on a specific diet, and 2.0% (*n* = 1) followed a protein-heavy or gluten-free diet. Furthermore, 50.0% (*n* = 25) reported using vitamin and mineral supplements, with the most commonly used being Vitamin D (28.6%, *n* = 18), iron (12.7%, *n* = 8), and Vitamin B12 (7.9%, *n* = 5) (Table 2)

^a^Pearson Chi-Square test, ^b^Fisher’s Exact test

*Since responses allowed for multiple answers, only n (%) values are provided

*p* < 0.05

When examining the effects of foods on symptoms, it was found that 82.0% of the patients (*n* = 41) had foods that exacerbated their symptoms, with the most common being legumes at 21.0% (*n* = 25), milk at 14.3% (*n* = 17), and raw vegetables at 10.9% (*n* = 13). Additionally, 50.0% of the participants (*n* = 25) reported foods that alleviated their symptoms, with the most common being yogurt 18.2% (*n* = 12) and cooked vegetables at 18.2% (*n* = 12) and cooked meat at 7.6% (*n* = 5) (Table 3).

**Table 3:** Assessment of Foods that Exacerbate or Alleviate Symptoms

Variables	IBD Diagnosis		
	Crohn’s Disease (*n* = 30)	Ulcerative Colitis (*n* = 20)	*p*
Are there any foods that alleviate symptoms?			
Yes	16 (53,3)	9 (45)	0.564^a^
No	14 (46,7)	11 (55)	
Types of foods that alleviate symptoms*			
No	15 (40,5)	10 (34,5)	**-**
Yogurt	8 (21,6)	4 (13,8)	
Cooked meat	1 (2,7)	4 (13,8)	
Banana	-	2 (6,9)	
Lactose-free milk	1 (2,7)	-	
Kefir	1 (2,7)	-	
Cooked vegetables	6 (16,2)	6 (20,7)	
Whole wheat bread	-	1 (3,4)	
Broccoli	1 (2,7)	-	
Cauliflower	2 (5,4)	2 (6,9)	
Other	2 (5,4)	-	
Are there any foods that exacerbate symptoms?			
Yes	24 (80)	17 (85)	0.724^b^
No	6 (20)	3 (15)	
Types of foods that exacerbate symptoms*			
Meat	2 (2,7)	1 (2,3)	-
Milk	10 (13,3)	7 (15,9)	
Legumes	17 (22,7)	8 (18,2)	
Raw vegetables	8 (10,7)	5 (11,4)	
Nuts	3 (4,0)	1 (2,3)	
Eggs	2 (2,7)	-	
Onion	2 (2,7)	-	
Yogurt	1 (1,3)	3 (6,8)	
Fatty foods	3 (4,0)	3 (6,8)	
Spices	7 (9,3)	4 (9,1)	
Coffee	7 (9,3)	1 (2,3)	
Kefir	-	1 (2,3)	
Tomato	7 (9,3)	-	
Apple	2 (2,7)	1 (2,3)	
Mandarin	2 (2,7)	-	
Fish	-	1 (2,3)v	
Orange	-	1 (2,3)	
Spicy foods	7 (9,3)	3 (6,8)	
Acidic foods	2 (2,7)	3 (6,8)	
Other	5 (6,7)	1 (2,3)	

a=Pearson Chi-Square test, b=Fisher’s Exact test

*Since responses allowed for multiple answers, only n(%) values are provided

*p* < 0.05

The mean daily energy intake was 1482.79 ± 545.90 kcal/day for women and 1765.02 ± 735.15 kcal/day for men. Mean daily fiber intake was 16.77 ± 7.55 g/day for women and 16.80 ± 8.05 g/day for men, both below recommended levels. Furthermore, intake was found to be below EFSA reference values for vitamin C, vitamin B1, vitamin B2, vitamin B6, folate, potassium, calcium, magnesium, and iron, based on a single 24-hour dietary recall (Table 4).

**Table 4:** Comparison of Food Consumption Record Scores by Gender (EFSA Recommendations)

Variables	Gender						
	Women		Men				Recommended (Women-Men)
	Median (IQR)	Mean ± SD	Median (IQR)	Mean ± SD	Z	*p*	-
Energy (kcal)	1401,1 (500,55)	1482,79 ± 545,9	1610,6 (1182,3)	1765,02 ± 735,15	-0.934	0.350	-
Protein (g)	59,1 (27,9)	61,49 ± 21,63	55,9 (43,9)	68,08 ± 27,9	-0.560	0.575	-
Fat (g)	71,2 (39,6)	71,55 ± 33,18	72,9 (41,75)	79,06 ± 38,23	-0.432	0.665	-
CHO (g)	126 (65,35)	145,42 ± 66,43	169,4 (125,8)	191,97 ± 95,8	-1.799	0.072	-
Fiber (g)	15,8 (9,05)	16,77 ± 7,55	14,5 (13,25)	16,8 ± 8,05	-0.187	0.852	25
Polyunsaturated fat (g)	10,2 (9,7)	11,71 ± 7,12	9,7 (10,35)	12,97 ± 8,07	-0.403	0.687	-
Vitamin A (µg)	713,5 (610,25)	815,83 ± 483,15	753,9 (544,5)	899,87 ± 591,99	-0.226	0.821	650–750
Vitamin E (mg)	8,2 (11,7)	11,85 ± 8,27	9,6 (8,25)	12,01 ± 7,79	-0.157	0.875	11–13
Vitamin B1 Thiamine (mg)	0,7 (0,4)	0,77 ± 0,36	0,7 (0,5)	0,8 ± 0,4	-0.01	0.992	1,1–1,2
Vitamin B2 Riboflavin (mg)	1,3 (0,55)	1,27 ± 0,47	1,2 (0,4)	1,24 ± 0,46	-0.217	0.828	1,6
Vitamin B6 Pyridoxine (mg)	0,9 (0,6)	0,91 ± 0,39	1 (0,6)	1,08 ± 0,52	-0.977	0.329	1,6 − 1,7
Folate (µg)	213 (119,55)	240,63 ± 109,77	233,6 (139,25)	242,38 ± 107,61	-0.128	0.898	330
Vitamin C (mg)	47,9 (60,3)	61,53 ± 45,15	40,7 (69,75)	52,07 ± 39,55	-0.816	0.415	95–110
Sodium (mg)	2537,6 (1822,35)	4330,4 ± 8380,48	3102 (2110,1)	3187,4 ± 1437,38	-1.111	0.267	2000
Potassium (mg)	1723,8 (659,2)	1760,47 ± 626,98	1855,2 (1039,35)	1901,51 ± 682,9	-0.816	0.415	3500
Calcium (mg)	589,4 (336,65)	592,9 ± 253,64	632,5 (453,6)	660,69 ± 269,37	-0.717	0.473	950
Magnesium (mg)	214,4 (94,15)	235,69 ± 111,22	227,5 (147,6)	236,07 ± 98,87	-0.226	0.821	300–350
Phosphorus (mg)	953,9 (428,3)	970,11 ± 357,22	982,1 (543,65)	1021,16 ± 363,36	-0.442	0.658	550
Iron (mg)	7,3 (3,65)	7,43 ± 2,59	8 (4)	7,73 ± 2,76	-0.442	0.658	16 − 11
Zinc (mg)	8,1 (4,95)	8,67 ± 3,42	10 (5,5)	10,15 ± 3,57	-1.366	0.172	7,5–12,7 / 9,4–16,3

Z=Mann Whitney U test

*p* < 0.05

A statistically significant difference was found between the GSRS Score and the meal skipping status (KW = 7.814, *p* = 0.020), where meal skipping was associated with higher gastrointestinal symptom burden. According to the results of the Games-Howell post-hoc analysis, a statistically significant difference was identified between meal skippers and non-skippers (*p* = 0.017), as well as between those who sometimes skipped meals and those who did not (*p* = 0.023). A statistically significant difference was also found between the meal skipping status and the subdimensions of the GSRS for “reflux syndrome” (KW = 6.802, *p* = 0.033) and “indigestion syndrome” (KW = 9.398, *p* = 0.009). Those who skipped meals had higher scores for “reflux” and “indigestion” compared to the other groups. According to the results of the Games-Howell post-hoc analysis, a statistically significant difference was identified between meal skippers and non-skippers (*p* = 0.035, *p* = 0.002), as well as between those who sometimes skipped meals and those who did not (*p* = 0.019, *p* = 0.027) (Table 5). Supplementary Table 1 presents data on the relationship between meal skipping and IBD diagnosis.

**Table 5:** Comparison of Patients’ Meal Skipping Status with Total and Subscale Score Means

Variables	Meal Skipping	*n*	Median (IQR)	Mean ± SD	KW	*p*	Post-Hoc
Beck Depression Inventory II	1)Yes	16	10,5 (8,75)	11,00 ± 6,53	3.216	0.200	-
	2)No	13	6 (7,5)	7,69 ± 6,94			
	3)Sometimes	21	7 (6)	9,29 ± 5,58			
IBD Quality of Life	1)Yes	16	183 (33)	184,69 ± 25,65	3.170	0.205	-
	2)No	13	214 (37,5)	200,08 ± 27,46			
	3)Sometimes	21	189 (29)	187,33 ± 28,46			
Gastrointestinal Symptom Rating Scale	1)Yes	16	44 (24,25)	44,44 ± 15,81	7.814	**0.020**	1 > 2 2 < 3
	2)No	13	28 (17)	29,92 ± 10,50			
	3)Sometimes	21	46 (23)	41,71 ± 13,86			
GSRS Abdominal Pain Syndrome	1)Yes	16	6 (5,25)	7,38 ± 4,98	2.610	0.271	-
	2)No	13	3 (4)	5,08 ± 2,84			
	3)Sometimes	21	5 (3)	5,67 ± 3,14			
GSRS Reflux Syndrome	1)Yes	16	4 (3,75)	4,38 ± 3,18	6.802	**0.033**	1 > 2 2 < 3
	2)No	13	2 (0)	2,15 ± 0,38			
	3)Sometimes	21	2 (3)	3,71 ± 2,37			
GSRS Diarrhea Syndrome	1)Yes	16	7 (7)	8,19 ± 6,15	3.768	0.152	-
	2)No	13	6 (5,5)	6,62 ± 3,93			
	3)Sometimes	21	11 (10,5)	10,52 ± 6,33			
GSRS Indigestion Syndrome	1)Yes	16	17 (9,25)	16,94 ± 5,87	9.398	**0.009**	1 > 2 2 < 3
	2)No	13	10 (7,5)	9,92 ± 4,19			
	3)Sometimes	21	15 (10,5)	14,76 ± 6,13			
GSRS Constipation Syndrome	1)Yes	16	7 (5,75)	7,56 ± 4,00	3.682	0.159	-
	2)No	13	3 (3)	6,15 ± 5,55			
	3)Sometimes	21	7 (6)	7,05 ± 4,36			

KW=Kruskal Wallis H test, Post-Hoc=Games-Howell

*p* < 0.05

In our study, a statistically significant positive correlation was found between BMI values and the “indigestion” scores from the GSRS (Rho = 0.401, *p* = 0.004). A statistically significant negative correlation was identified between the duration of IBD diagnosis and BDI scores (Rho=-0.288, *p* = 0.047), while a positive correlation was found between the duration of IBD diagnosis and the “reflux” scores from the GSRS (Rho = 0.371, *p* = 0.009). There was a statistically significant negative correlation between the BDI score and the total score of the IBD-QoL scale (Rho=-0.630, *p* < 0.001), whereas a positive correlation was found between the BDI score and the GSRS score (Rho = 0.286, *p* = 0.044). Additionally, a statistically significant positive correlation was found between the BDI score and the GSRS subdimensions of “abdominal pain” (Rho = 0.295, *p* = 0.037), “indigestion” (Rho = 0.279, *p* = 0.049), and “constipation” (Rho = 0.386, *p* = 0.006). A statistically significant negative correlation was found between the IBD-QoL score and the GSRS score (Rho=-0.720, *p* < 0.001), as well as between the IBD-QoL score and the GSRS subdimensions of “abdominal pain” (Rho=-0.559, *p* < 0.001), “diarrhea” (Rho=-0.547, *p* < 0.001), “indigestion” (Rho=-0.514, *p* < 0.001), and “constipation” (Rho=-0.477, *p* < 0.001). Furthermore, a statistically significant negative correlation was observed between the IBD-QoL subdimension of “bowel symptoms” and the GSRS score (Rho=-0.341, *p* = 0.015), as well as between the IBD-QoL subdimension of “bowel symptoms” and the GSRS subdimension of “constipation” (Rho=-0.315, *p* = 0.026). Finally, a statistically significant negative correlation was found between the IBD-QoL subdimension of “emotional function” and the GSRS subdimension of “reflux” (Rho=-0.310, *p* = 0.029) (Table 6).

**Table 6:** Correlation Results of the Relationship Between Patients’ Total and Subscale Scores and Various Variables

		1	2	3	4	5	6	7	8	9	10	11	12	13	14	15	16	17
1-Age	Rho	1																
	p	.																
2-BMI	Rho	,532**	1															
	p	**< 0.001**	.															
3-IBD Diagnosis Duration (years)	Rho	,424**	0,098	1														
	p	**0**,**003**	0,507	.														
4-Daily Sleep Duration	Rho	0,03	0,127	0,023	1													
	p	0,839	0,381	0,875	.													
5-Beck Depression Inventory II	Rho	-0,258	-0,147	-,288*	0,148	1												
	p	0,07	0,307	**0**,**047**	0,306	.												
6-IBD Quality of Life	Rho	0,122	0,109	0,058	-0,108	-,630**	1											
	p	0,398	0,45	0,696	0,455	**< 0.001**	.											
7-IBD Systemic Symptoms	Rho	0,084	-0,182	0,184	-0,111	0,038	0,013	1										
	p	0,564	0,207	0,21	0,443	0,794	0,929	.										
8-IBD Bowel Symptoms	Rho	-0,044	-0,034	-0,089	-0,061	-0,047	,298*	,623**	1									
	p	0,763	0,816	0,549	0,676	0,746	**0**,**035**	**< 0.001**	.									
9-IBD Functional Impairment	Rho	0,172	0,113	0,016	0,032	0,093	-0,092	,590**	,577**	1								
	p	0,233	0,434	0,912	0,825	0,522	0,523	**< 0.001**	**< 0.001**	.								
10-IBD Social Impairment	Rho	-0,082	-0,158	0,137	0,062	0,054	-0,051	,540**	,496**	,599**	1							
	p	0,569	0,272	0,354	0,671	0,712	0,726	**< 0.001**	**< 0.001**	**< 0.001**	.							
11-IBD Emotional Function	Rho	-0,085	-0,167	-0,073	-0,169	-0,13	0,081	,395**	,373**	,338*	,440**	1						
	p	0,559	0,247	0,624	0,241	0,37	0,574	0,005	**0**,**008**	**0**,**016**	**0**,**001**	.						
12-GSRS Symptom Rating Scale	Rho	0,068	0,19	0,068	0,018	,286*	-,720**	-0,193	-,341*	-0,125	-0,130	-0,155	1					
	p	0,641	0,186	0,648	0,900	**0**,**044**	**< 0.001**	0,180	**0**,**015**	0,389	0,369	0,281	.					
13-GSRS Abdominal Pain Syndrome	Rho	-0,192	-0,129	-0,017	0,032	,295*	-,559**	-0,145	-0,205	-0,103	-0,067	0,046	,682**	1				
	p	0,182	0,372	0,91	0,826	**0**,**037**	**< 0.001**	0,314	0,153	0,476	0,642	0,751	**< 0.001**	.				
14-GSRS Reflux Syndrome	Rho	0,241	0,177	,371**	0,088	0,149	-0,226	0,052	-0,084	-0,034	-0,162	-,310*	,443**	0,138	1			
	p	0,092	0,219	**0**,**009**	0,545	0,302	0,115	0,720	0,564	0,817	0,261	**0**,**029**	**0**,**001**	0,34	.			
15-GSRS Diarrhea Syndrome	Rho	-0,057	0,121	0,103	0,042	0,163	-,547**	-0,200	-0,24	-0,016	0,055	-0,077	,710**	,497**	0,193	1		
	p	0,694	0,403	0,485	0,771	0,257	**< 0.001**	0,164	0,093	0,913	0,704	0,593	**< 0.001**	**< 0.001**	0,179	.		
16-GSRS Indigestion Syndrome	Rho	0,247	,401**	0,029	0,129	,279*	-,514**	-0,006	-0,13	-0,037	-0,093	-0,166	,770**	,383**	,508**	,281*	1	
	p	0,084	**0**,**004**	0,845	0,373	**0**,**049**	**< 0.001**	0,970	0,368	0,799	0,522	0,248	**< 0.001**	**0**,**006**	**< 0.001**	**0**,**048**	.	
17-GSRS Constipation Syndrome	Rho	-0,101	0,096	-0,131	-0,077	,386**	-,477**	-0,259	-,315*	-0,172	-0,159	-0,031	,594**	,434**	0,049	,331*	,301*	1
	p	0,485	0,506	0,373	0,596	**0**,**006**	**< 0.001**	0,070	**0**,**026**	0,232	0,271	0,829	**< 0.001**	**0**,**002**	0,733	**0**,**019**	**0**,**034**	.

*Correlation is significant at the 0.05 level (Spearman correlation test), **Correlation is significant at the 0.01 level (Spearman correlation test)

## Discussion

IBD, defined as a chronic and recurrent inflammatory disorder, significantly affects quality of life and leads to complications. Nutrition plays a crucial role in IBD management. Understanding patients’ dietary behaviors and their impact on disease progression is therefore critical for effective clinical care [9].

Identifying dietary habits and understanding their effects on IBD disease course is of great clinical importance. Studies on meal skipping and eating patterns have shown that meal timing and frequency are associated with many chronic diseases, particularly metabolic syndrome [38]. A systematic review of 35 articles investigating the effects of meal skipping found that breakfast is the most commonly skipped meal among young adults aged 18 to 30 years [39]. In the present study, the most commonly reported reasons for meal skipping were time constraints (46.7% in CD, 60.0% in UC), followed by symptoms (13.3% in CD, 5.0% in UC) and lack of appetite (10.0% in CD, 5.0% in UC), suggesting that both lifestyle and disease-related factors contribute to irregular meal patterns in IBD patients (Supplementary Table 1). There is a relationship between the number of meals consumed daily and chronic diseases. In a study examining meal frequency and patterns, increasing the number of meals and eating more frequently was associated with lower obesity rates compared to consuming fewer than three meals a day [40]. A study conducted in Sweden found that irregular eating patterns were associated with an increased risk of cardiovascular disease in men [41]. In our study, 24.0% (*n* = 12) of patients reported regular meal consumption without skipping, 44.0% (*n* = 22) sometimes skipped meals, and 32.0% (*n* = 16) skipped meals regularly. The most frequently skipped meal was lunch, with a prevalence of 56.0% (*n* = 28). In a study conducted in Jordan involving 185 IBD patients and 150 controls, the most commonly skipped meal was dinner [42], which differs from the findings of the present study.

Food choices are a primary concern for patients with IBD [43]. Dietary habits and beliefs are influenced by the relationship with foods and can become ingrained due to misinformation or individual carelessness [44]. A study conducted in the UK found that 49.0% of IBD patients avoided certain foods [45]. In a study conducted in France, 47.5% (*n* = 116) of patients reported that their taste preferences had changed, and 66.8% (*n* = 163) stated that they no longer consumed certain foods they enjoyed to prevent disease recurrence [46]. According to a study conducted in the Netherlands, which evaluated beliefs and behaviors related to nutrition among 294 IBD patients, 65.2% (*n* = 191) believed that eating regular meals was effective in reducing disease symptoms [47].

In our study, the majority of patients, 82.0% (*n* = 41), reported that certain foods exacerbated their symptoms. The foods that worsened symptoms included legumes (21.0%), milk (14.3%), raw vegetables (10.9%), spices (9.2%), spicy foods (8.4%). Half of the patients (*n* = 25) indicated that there were foods that alleviated their symptoms. The foods that reduced symptoms included yogurt (18.2%), cooked vegetables (18.2%), cooked meat (7.6%), cauliflower (6.1%). A study conducted in the USA involving 2,329 IBD patients reported that vegetables, spicy foods, fruits, nuts, fried foods, milk, red meat, soda, popcorn, dairy products, alcohol, high-fiber foods, corn, fatty foods, seeds, and coffee exacerbated symptoms, whereas yogurt, bananas, and rice improved symptoms [48]. In a study conducted in Ireland, which examined 475 IBD patients, 73.0% of patients reported eating the same meals as other individuals living in their households, 56.0% avoided eating out due to fear of disease recurrence or exacerbation, and 70.0% avoided foods and beverages they enjoyed [49]. The foods most often reported to worsen or alleviate symptoms in the present study are broadly consistent with findings from these previous studies, suggesting that dietary patterns and individualized meal planning are important considerations in IBD management.

Food consumption records are comprehensive accounts of all foods, beverages, and dietary supplements consumed by individuals over a specific period [50]. In our study, dietary intake was assessed using a 24-hour dietary recall, which was analyzed using the BeBiS dietary analysis software and compared against reference values recommended by the EFSA [28, 29]. It should be noted that a single 24-hour recall may not accurately reflect habitual dietary intake due to day-to-day variability; therefore, the following findings should be interpreted as estimates of intake on the recall day rather than as definitive assessments of habitual nutrition. The average daily energy intake for IBD patients in our study was found to be 1482.79 ± 545.90 kcal/day for women and 1765.02 ± 735.15 kcal/day for men. When compared against EFSA recommended amounts, the average daily energy intakes observed in our study were lower than reference values. It is suggested that healthy adults obtain 45–60% of their energy from carbohydrates, 10–20% from proteins, and 20–35% from fats [29]. The low-calorie intake observed in our data may be attributed to factors such as individuals restricting food intake due to symptoms, avoiding various foods out of fear of exacerbating their condition, having poor dietary habits due to misinformation, and failing to obtain energy from appropriate sources. The data obtained from our study supports this observation, as it highlights the participants’ meal skipping and food restrictions. In our study, it was determined that women consumed 16.77 ± 7.55 g of fiber daily, while men consumed 16.80 ± 8.05 g. These findings are consistent with previous research indicating that IBD patients consume significantly less fiber than healthy controls, even when clinical remission is achieved and no symptoms dictate dietary restrictions [51]. This suggests that low fiber intake may be a persistent dietary habit in IBD patients, possibly driven by psychological barriers or learned food avoidance rather than active disease status. A study on dietary fiber found that patients consuming a diet with 24.3 g/day of fiber had a 40% reduction in the risk of developing CD, while no relationship was observed for UC [52]. Although many IBD patients tend to avoid fiber-containing foods due to their effects on symptoms, this can lead to micronutrient deficiencies and exacerbate malnutrition [15].

The most common micronutrient deficiencies observed in patients with IBD are vitamin D, folate, and iron [22]. A study examining the food consumption of 84 patients with CD and 42 patients with UC in the USA found that they were insufficient in vitamins A, D, E, C, calcium, folate, and iron [53]. According to another study conducted in the USA involving 12 UC and 46 CD patients, individuals were found to be deficient in vitamins A, D, E, and zinc [54]. In our study, intake was found to be below EFSA dietary reference values for vitamin C, vitamin B1, vitamin B2, vitamin B6, folate, potassium, calcium, magnesium, and iron based on the single 24-hour dietary recall. These findings are consistent with the broader literature and underscore the importance of routine nutritional screening and individualized dietary support in IBD management.

Quality of life is defined as an individual’s perception of their position in life in relation to their concerns, goals, and expectations [55]. In patients with IBD, symptoms can lead to significant changes that impact not only physical, emotional, and social aspects but also attitudes, behaviors, and productivity [56]. A study conducted in France examining the impact of IBD on social life reported that 21.7% of 244 IBD patients (*n* = 53) refused to eat out due to fear of relapse. The necessity to eliminate many foods from the diet also complicates dining out. It was found that nearly one in five IBD patients (*n* = 47) could not share the same menu with other family members [44]. A study conducted in the UK involving 1,221 patients aimed to assess food-related quality of life in individuals with IBD and concluded that the low quality of life specific to IBD is associated with poorer dietary quality [57]. In our study, a statistically significant negative relationship was identified between the IBD-QoL score and the GSRS score, as well as between the GSRS subdimensions of “abdominal pain,” “diarrhea,” “indigestion,” and “constipation” (*p* < 0.001). Higher GSRS scores were associated with lower IBD-QoL scores. Additionally, a statistically significant negative relationship was found between the IBD-QoL subdimension of “bowel symptoms” and the GSRS score (*p* = 0.015), as well as between the GSRS subdimension of “constipation” (*p* = 0.026). Higher GSRS scores were also associated with lower scores for the “bowel function” subdimension of the IBD-QoL. A statistically significant difference was found between the type of IBD diagnosis and the IBD-QoL subdimension of “systemic symptoms.” The “systemic symptoms” score was higher in UC patients compared to CD patients (*p* = 0.049). It should be noted, however, that disease activity indices (e.g., Mayo score, CDAI), medication use, and other clinical variables were not assessed. Therefore, residual confounding due to unmeasured disease activity cannot be excluded, and the observed associations should be interpreted with caution. Nevertheless, literature suggests that dietary patterns in IBD remain altered regardless of clinical status. For instance, a study demonstrated that even patients in drug-induced clinical remission exhibit different macronutrient and fiber intakes compared to healthy subjects [51]. Furthermore, research specifically comparing active and quiescent Crohn’s disease patients (*n* = 117) has shown that nutrient intake remains similar regardless of clinical disease activity, with both groups failing to meet recommended values for fiber (mean intake below 25 g/day) and various micronutrients, including potassium, calcium, magnesium, and vitamins [58]. This evidence suggests that nutritional inadequacies in IBD may persist independently of clinical disease phase, providing some contextual support for examining dietary behaviors as a distinct area of investigation.

Depression, a prevalent mental disorder worldwide, is characterized by a depressive mood along with anhedonia or loss of interest in activities [59]. Patients with IBD are significantly exposed to anxiety and depression symptoms, with findings indicating that approximately one in three patients experiences anxiety symptoms, and one in four patients suffers from depression symptoms [60]. Our focus on mental health in this population is strongly supported by a large-scale meta-analysis with 30,118 IBD patients, which reported a pooled prevalence of 25.2% for depression symptoms, highlighting that psychological distress is a core component of the disease burden [61]. Compared to the general population, IBD patients face a twofold higher risk of major depression [59]. In our study, a statistically significant negative association was found between the duration of IBD diagnosis and BDI scores (*p* = 0.047), suggesting that longer disease duration is associated with lower depression scores. This may reflect a process of gradual psychological adaptation to living with a chronic illness over time. Consistent with our observations, a major systematic review highlighted that quality of life in IBD patients tends to improve over time, suggesting that patients may develop better resilience and coping strategies as they navigate the disease course [62].

To our knowledge, no studies in the literature have specifically examined the relationship between meal patterns and gastrointestinal symptoms in IBD patients. A study conducted on Irritable Bowel Syndrome (IBS) involving 4,599 adults from 50 different healthcare centers in Iran found that participants who consumed meals regularly experienced a decrease in both the risk of developing IBS and the severity of symptoms compared to those who did not eat regularly or skipped meals [63]. In a study conducted in Japan with 294 patients with UC, the dietary attitudes and behaviors of patients were investigated, revealing that those who skipped breakfast had lower mucosal healing compared to those who did not skip this meal [64]. In our study, meal skipping was significantly associated with higher GSRS scores (*p* = 0.020), with post-hoc analysis confirming significant differences between meal skippers and non-skippers (*p* = 0.017) and between occasional meal skippers and non-skippers (*p* = 0.023). Meal skipping was also significantly associated with higher scores for reflux (*p* = 0.033) and indigestion (*p* = 0.009) subdimensions of the GSRS. These findings suggest an association between irregular meal patterns and greater gastrointestinal symptom burden in IBD patients. However, reverse causality cannot be excluded, and residual confounding due to unmeasured disease activity may have influenced the observed associations.

### Strengths of the study

This multifaceted pilot study evaluates individuals from multiple perspectives, including dietary intake, meal patterns, gastrointestinal symptoms, quality of life, and depression, providing a comprehensive view rarely captured in a single assessment. The use of internationally recognized and validated instruments, such as the IBD-QoL, BDI-II, and GSRS, significantly enhances the reliability and comparability of the findings. Furthermore, to our knowledge, this study is among the first to specifically examine the association between meal skipping and gastrointestinal symptom burden in IBD patients, addressing a clinically relevant but previously understudied area of patient behavior. By focusing on these dietary and behavioral factors in a real-world outpatient setting, the study provides an exploratory perspective on how patients’ daily habits correlate with their perceived symptom burden.

### Limitations of the study

Several limitations must be acknowledged when interpreting these results. First, the relatively small sample size and the single-center design, with recruitment from a single outpatient clinic, may limit the generalizability of the findings to a broader IBD population. Additionally, analyzing Crohn’s disease and Ulcerative colitis patients together may have obscured certain disease-specific differences. Second, formal disease activity indices, specific medication use (including biologics, steroids, and immunomodulators), and surgical history were not systematically collected. These factors represent important potential confounders for gastrointestinal symptoms and quality of life that should be incorporated into future research. In addition, due to the limited sample size and absence of disease activity measures, multivariate regression analyses were not performed, and residual confounding cannot be excluded. Third, due to the cross-sectional design, it is difficult to determine whether the observed dietary patterns and meal skipping behaviors are a cause or a consequence of symptom severity; therefore, reverse causality cannot be excluded. Finally, nutritional and anthropometric data were self-reported and based on a single 24-hour dietary recall, which is subject to recall bias and may not fully reflect habitual intake due to day-to-day variability. Larger, long-term prospective studies are required to confirm and expand upon these findings.

## Conclusion

Identifying dietary habits is crucial for disease management and symptom control in patients with IBD. Eliminating certain foods from the dietary pattern may lead to micronutrient deficiencies in the body. Therefore, a personalized nutrition plan should be developed for patients, taking into account their individual dietary needs as well as their experiences with foods that exacerbate or alleviate symptoms and potentially influence disease progression. In addition, the observed association between higher depression levels, increased gastrointestinal symptoms, and poorer quality of life highlights the importance of a multidisciplinary approach in IBD management. Regular eating patterns and avoidance of meal skipping were associated with lower gastrointestinal symptom scores in the present study, though the direction of this relationship warrants further investigation. Our findings suggest that meal patterns and dietary habits are associated with gastrointestinal symptoms and quality of life in patients with IBD. However, due to the cross-sectional design, causal relationships cannot be established. Future prospective studies incorporating disease activity indices, medication use, and longer follow-up periods are needed to better clarify these associations and to confirm the clinical relevance of meal regularity in IBD management.

## Supplementary Information

Supplementary Material 1.

Supplementary Material 2.

Supplementary Material 3.

## Data Availability

The datasets generated and analyzed during this study are available from the corresponding author upon reasonable request. This study was registered as a clinical trial on Clinical Trials (Trial Registration Number: NCT07179965; Date of Registration: August 2023).
