# Supplementary material for: Associations of dietary habits and meal regularity with gastrointestinal symptoms, depression, and quality of life in patients with inflammatory bowel disease: a cross-sectional study
Source: BMC Gastroenterol. 2026 Jun 17;26:529. doi: 10.1186/s12876-026-04999-9 (PMC13520149; doi:10.1186/s12876-026-04999-9)
Supplement: Supplementary file 1 [file 12876_2026_4999_MOESM2_ESM.pdf]

Submission ID: 893ef78f-295e-4762-a2a4-0a994948d358
Corresponding author: Elif Nur ÖZEN, elifnur.ozen@istinye.edu.tr

24-HOUR DIETARY RECALL

Meal Time	Food Items and Ingredients	Amount (household measures)
**Breakfast**		
**Morning Snack**		
**Lunch**		
**Afternoon Snack**		
**Dinner**		
**Night Snack**		
