# Supplementary material for: Associations of dietary habits and meal regularity with gastrointestinal symptoms, depression, and quality of life in patients with inflammatory bowel disease: a cross-sectional study
Source: BMC Gastroenterol. 2026 Jun 17;26:529. doi: 10.1186/s12876-026-04999-9 (PMC13520149; doi:10.1186/s12876-026-04999-9)
Supplement: Supplementary file 3 [file 12876_2026_4999_MOESM1_ESM.docx]

**SUPPLEMENTARY MATERIALS**

**Table 1:** Evaluation of Meal Skipping in Relation to IBD Diagnosis

| **Variables** | **IBD Diagnosis** | |  |
| --- | --- | --- | --- |
|  | **Crohn's Disease (n=30)** | **Ulcerative Colitis (n=20)** | **p** |
| **Number of Main Meals** |  |  |  |
| 1 meal | 1 (3,3) | 0 (0) | 1.000^b^ |
| 2 meals | 11 (36,7) | 7 (35) |  |
| 3 meals | 18 (60) | 13 (65) |  |
| **Number of Snacks** |  |  |  |
| None | 15 (50) | 4 (20) | 0.081^b^ |
| 1 meal | 11 (36,7) | 11 (55) |  |
| 2 meals | 3 (10) | 5 (25) |  |
| 3 meals | 1 (3,3) | 0 (0) |  |
| **Is a Meal Skipped?** |  |  |  |
| Yes | 12 (40) | 4 (20) | 0.240^a^ |
| No | 8 (26,7) | 4 (20) |  |
| Sometimes | 10 (33,3) | 12 (60) |  |
| **Which Meal is Skipped?** |  |  |  |
| None | 8 (26,7) | 4 (20) | 0.674^b^ |
| Breakfast | 6 (20) | 3 (15) |  |
| Lunch | 15 (50) | 13 (65) |  |
| Dinner | 1 (3,3) | 0 (0) |  |
| **Reason for Skipping Meals** |  |  |  |
| No meal skipped | 8 (26,7) | 4 (20) | 0.704^b^ |
| Time constraints | 14 (46,7) | 12 (60) |  |
| Lack of appetite | 3 (10) | 1 (5) |  |
| Not a habit | 1 (3,3) | 1 (5) |  |
| Due to snacking | 0 (0) | 1 (5) |  |
| Due to symptoms | 4 (13,3) | 1 (5) |  |
| **Do Symptoms Affect the Number of Meals?** |  |  |  |
| Yes | 13 (43,3) | 9 (45) | 0.907^a^ |
| No | 17 (56,7) | 11 (55) |  |

**a=Pearson Chi-Square test, b=Fisher’s Exact test, p<0.05**

It was found that 62.0% (n=31) of the patients consumed three main meals, 44.0% (n=22) had one snack, 44.0% (n=22) sometimes skipped meals, 56.0% (n=28) skipped the lunch meal, and 52.0% (n=26) skipped meals due to time constraints. Additionally, 44.0% (n=22) reported that their symptoms affected their meal frequency (Supplementary Table 1).
